# Paleontology in the 21st Century

**DOI:** 10.3390/biology12030487

**Published:** 2023-03-22

**Authors:** Mary H. Schweitzer

**Affiliations:** 1 Department of Biological Sciences, North Carolina State University, Raleigh, NC 27606, USA; schweitzer@ncsu.edu; 2 North Carolina Museum of Natural Sciences, Raleigh, NC 27601, USA; 3 Department of Geology, Lund University, 223 62 Lund, Sweden; 4 Museum of the Rockies, Montana State University, Bozeman, MT 59717, USA

For much of its 300+ year history, “modern” paleontology has been a descriptive science, firmly housed within geological sciences. The application of rigorous phylogenetic methods [1,2,3] to extinct organisms was a driver of a movement that began in the 1980s to bring paleontology, and particularly deep time paleontology (i.e., >1 Ma), more firmly into the biological sciences. Paleohistology—the investigation of the microstructure of fossil bones initiated by Enlow [4,5] and expanded upon by De Ricqlés and colleagues—(e.g., [5,6,7]) also influenced how dinosaurs and other fossils would be studied, as biological organisms rather than as geological oddities. The microscopic examination of ancient bone—even dinosaur bone—has revealed the presence of osteons, osteocyte lacunae, and vascular canals in these ancient specimens. Rather than being obliterated by fossilization processes, the retention of these features in bone recovered from Mesozoic and earlier sediments has allowed us to discern biological information from these once-living organisms, such as comparative growth rates and estimates of ontogenetic stages [7,8,9]. Phylogenetic and histological analyses forever changed the direction of paleontological studies.

However, the application of most molecular biological tools to elucidate evolutionary processes and timing was, until recently, reserved for extant species. The genomic revolution fostered by the advent of a polymerase chain reaction (PCR) and next gen technologies was limited in application to only living or very recently extinct organisms, and was not rigorously and regularly applied to deep time fossils, Jurassic Park notwithstanding. 

Although DNA technologies were more readily accepted and proved useful for archaeological materials (see, e.g., [10,11,12,13]), the assumption that original, informative organic components were lost at some point during the transition from the biosphere to the geosphere [14,15], and the proposal of a predictable, and short, half-life for DNA and other biomolecules effectively slowed the application of these methods to all but the most recent fossil materials. What else older fossils may be telling us, and what else was possible to know, were questions that were not widely asked.

This is beginning to change, as illustrated in this Special Issue, because it is widely recognized that 1. some endogenous structures and the molecules comprising them are retained in ancient specimens; 2. morphological studies alone have failed to adequately account for convergence or parallel evolution; and 3. fossils are absolutely necessary to incorporate into any studies seeking to determine the patterns, processes, and timing of evolution deep in the Earth’s history.

Just as technology in the life sciences has expanded at a record pace, the application of new technologies to paleontological specimens, though slower in coming, is resulting in an explosion in the type of data recoverable from fossils, as well as the type and age of fossils to which these can be applied. For example, the upper limit of DNA preservation, just 20 years ago, was proposed to be 100,000 years [10], but this limit has been repeatedly pushed back in time, most recently to >2 Ma with the recovery of environmental DNA from Greenland [16]. Protein sequence data, on the other hand, has been reported from 3 Ma eggshells [17] to multi-million-year-old dinosaurs [18,19], and immunological evidence for such preservation, suggested as far back as the 1950s [20,21], is becoming more prevalent. 

This Special Issue sheds light on the quantum leap in understanding the Earth’s rich biological history over the last 4 billion years, brought about by the application of new technologies to old fossils. It highlights new questions we can ask of the fossil record, and the expansion of fossils we can interrogate to yield robust high-resolution data. The authors within this Issue discuss the rapid development of new (or, new to paleontology) methods, once limited to extant organisms, that are now becoming broadly applied to fossils, including those from deep time. 

This Issue contains several broad review articles. Tihelka et al. [22] discuss the possibility that animals, in the form of arthropods, invaded the land several million years before what was previously assumed, yielding a terrestrial “Cambrian explosion” of these widespread and taxonomically diverse invertebrates. This hypothesis is supported by combining molecular clock data with fossil evidence. The discrepancies between molecular clock and fossil data are addressed, and a method combining molecular and morphological data to estimate lineage divergence in a total evidence framework is proposed.

The value of fossils for phylogenetic studies has always been recognized, but the value of fossils for molecular studies has recently, and increasingly, been elucidated by authors contributing here [23] and elsewhere. Torres et al. [24] review the long history of paleoimmunological approaches to paleontology, and apply these methods to fossils from the 1.3 Ma Venta Micena site, whereas Tahoun et al. [25] provide an overview of organic molecules recovered from non-avian dinosaurs and contemporary organisms, including pigments and various proteins. They highlight the effort within the paleontological community to understand the mechanisms of such preservation, contributing to the emerging disciplines of molecular paleontology, and molecular taphonomy/diagenesis. 

López-Antoñanzas and colleagues [26] further emphasize the need for incorporating fossil data to better understand evolutionary rates and relationships in their review of new, integrative phylogenetic methods. They stress the need for a unified approach to improve accuracy in modeling evolutionary processes and diversity distributions in deep time, and highlight new methods that combine morphological data from living and extinct groups with available molecular data to achieve more accurate evolutionary syntheses. Some of these include combining geometric morphometric and phylogenetic methods, incorporating stratigraphic data into parsimony analyses, and various statistical methods to calibrate a “morphological clock” that uses morphological data from both extant and extinct species. 

Tamborini [27] reviews the changing role of paleobiology between the 20th and 21st centuries, pointing to the necessity of fossils in elucidating “deep time patterns and processes”. He contrasts the historical differences between paleontology, a geology-based discipline, and the more biological approaches of paleobiology. He focuses his discussion first on the role of paleocolor as a testable hypothesis, stimulated by integrating more data with more technology; second, the search for endogenous organics in fossil materials that has driven the application of new (to paleontology) technologies, such as high-resolution, high-mass accuracy tandem mass spectrometry to address and characterize organic molecules in fossils; third, the integration of morphology and evolutionary theory in investigations of locomotion and mastication via the new role of robotics in 21st century; fourth, the relationship between evolution as expressed in fossils and the development of living organisms (‘evo-devo’), resulting in a broader integration between paleontology and biology; and finally, the examination of both biotic and abiotic factors in shaping organisms through evolution. He ends his review by encouraging a new synthesis of knowledge brought about by new data, new fossils, new technologies, and the deeper integration of these disciplines. 

Zhou [28] reviews a century of development in paleontology in China, identifying the influences of foreign collectors shaping the first part of this century-wide overview, and notes their influence of both geology and paleontology in China for decades. He notes not only the increasing participation of Chinese paleontologists to the discipline, but also the role of Chinese fossils, from early hominins (e.g., Peking Man, *Homo erectus*) to early feathered dinosaurs, in shaping a much broader picture of Earth’s history. Native-born Chinese paleontologists greatly expanded the breadth of paleontology in the 20th century and continue this trend today, both within and outside of China. The role of international collaborations, increased funding from government sources, and, of course, the incredibly rich paleontological flora and fauna in Chinese deposits has greatly expanded paleontology of all kinds, and will, no doubt, continue this trend into the foreseeable future.

Monson and colleagues [23] highlight various modern approaches to traditional paleontological questions, pointing out the expansion of data derived from CT imaging (including synchrotron imaging) to achieve 3D reconstruction of fossil data, and non-destructive means to study fossil histology as well. These authors describe how combining quantitative genetics and developmental biology approaches allows us to incorporate genotype:phenotype mapping to address morphological variation and its significance in informing phylogenetic history and the role of selection, and provide a case study using these tools.

The presence of endogenous molecules, including protein, DNA, various pigments, and biomarkers, is becoming an increasingly important aspect of paleontological studies, and new technology continues to push back the time limit for the preservation of these important molecules. New methods are being used to test hypotheses rooted in analyses of DNA. Churchill et al. [29] seeks to test the idea of interbreeding between Neandertal and modern humans in Europe and Asia, proposed in earlier DNA studies [30,31] by comparing facial size and shape parameters that may reflect the expression of Neandertal genes, using morphometric techniques.

A wide array of these new technologies are applied to address biomolecular remnants in bones as young as 350 years [32] and as old as the Carboniferous [33]. Phylogenetically and physiologically informative tissues were probed by synchrotron [34] to support the previous identification of reproductive tissues in dinosaurs [35,36]. Technologies continue to broaden not only the type of questions to be asked, but the type of fossils we can analyze, from coprolites [33], teeth [37], and invertebrates [22,38,39] to dinosaurs [25,34,40,41,42,43,44], mammals [45], and our own lineage [29,32,46].

Finally, taphonomic reconstructions remain an important part of paleobiology, and this Special Issue includes multi-dimensional studies on taphonomy using rare earth element studies (REE) to trace the movement of pore waters through bone during fossilization to elucidate the mechanisms contributing to molecular preservation in various dinosaur bone and other fossils [40,41,42,43]. However, the recovery of proteins also requires a better understanding of taphonomic modifications, as noted in [37] and previously discussed in [47].

Actualistic taphonomy experiments inform on the modifications introduced during diagenesis, but also informs on possible preservation conditions, recognizing that Mesozoic conditions were very different than today, and may have facilitated preservation through an elevated microbial response to high atmospheric CO_2_ [48]. Additionally, although exceptionally preserved tissues have long been the target of molecular studies on fossils, there is clearly more to the story, as illustrated by Colleary et al. [44], as not all exceptional fossils preserve endogenous biomolecules. 

This Special Issue briefly reviews these cutting-edge technologies and their applications to fossil data in various case studies, indicating a new ‘fossil renaissance’ for understanding life on this planet, yielding robust data that may be applied to understanding where we are going by better understanding from where we have come.

## References

[1] Padian K., Chiappe L.M. (1998). The origin and early evolution of birds. Biol. Rev..

[2] Gauthier J., Padian K. (1986). Saurischian monophyly and the origin of birds. The Origin of Birds and the Evolution of Flight.

[3] Padian K., Lindberg D.R., Polly P.D. (1994). Cladistics and the fossil record: The uses of history. Ann. Rev. Earth Planet. Sci..

[4] Enlow D.H., Brown S.O. (1958). A comparative histological study of fossil and recent bone tissues, Part I. Tex. J. Sci..

[5] Reid R.E.H. (1985). On supposed Haversian bone from the hadrosaur Anatosaurus, and the nature of compact bone in dinosaurs. J. Paleontol..

[6] De Ricqles A.J., Squelettiques E.F., Ura W.R.S.I., Via U.P., Padian K., Horner J.R., Francillon-vieillot H. (2000). Palaeohistology of the bones of pterosaurs and biomechanical implications. Zool. J. Linn. Soc..

[7] Horner J.R., Padian K., de Ricqlès A., de Ricqles A. (2001). Comparative osteohistology of some embryonic and perinatal archosaurs: Developmental and behavioral implications for dinosaurs. Paleobiology.

[8] De Ricqles A., Grupe G., Garland A.N. (1993). Some Remarks on Palaeohistology from a Comparative Evolutionary Point of View. Histology of Ancient Human Bone.

[9] De Ricqles A.J., Thomas D.K., Olson E.C. (1980). Tissue structures of dinosaur bone: Functional significance and possible relation to dinosaur physiology. A Cold Look at the Warm-Blooded Dinosaurs.

[10] Höss M. (2000). Neanderthal population genetics. Nature.

[11] Noro M., Masuda R., Dubrovo I.A., Yoshida M.C., Kato M. (1998). Molecular phylogenetic inference of the woolly mammoth Mammuthus primigenius, based on complete sequences of mitochondrial cytochrome b and 12S ribosomal RNA genes. J. Mol. Evol..

[12] Knapp M., Hofreiter M. (2014). Ancient Human DNA: Phylogenetic Applications. eLS.

[13] Hofreiter M., Collins M., Stewart J.R. (2012). Ancient biomolecules in Quaternary palaeoecology. Quat. Sci. Rev..

[14] Lindahl T. (1993). Instability and Decay of the Primary Structure of DNA. Nature.

[15] Lindahl T. (1997). Facts and artifacts of ancient DNA. Cell.

[16] Kjaer K.H., Pedersen M.W., De Sanctis B., De Cahsan B., Michelsen C.S., Sand K.K., Jelavić S., Ruter A.H., Bonde A.M.Z., Kjeldsen K.K. (2022). A 2-Million-year-old ecosystem in Greenland uncovered by Environmental DNA. Nature.

[17] Demarchi B., Hall S., Roncal-Herrero T., Freeman C.L., Woolley J., Crisp M.K., Wilson J., Fotakis A., Fischer R., Kessler B.M. (2016). Protein sequences bound to mineral surfaces persist into deep time. Elife.

[18] Cleland T.P., Schroeter E.R., Zamdborg L., Zheng W., Lee J.E., Tran J.C., Bern M., Duncan M.B., Lebleu V.S., Schweitzer M.H. (2015). Mass spectrometry and antibody-based characterization of blood vessels from Brachylophosaurus canadensis. J. Proteome Res..

[19] Schroeter E.R., Dehart C.J., Cleland T.P., Zheng W., Thomas P.M., Kelleher N.L., Bern M., Schweitzer M.H. (2017). Expansion for the Brachylophosaurus canadensis Collagen i Sequence and Additional Evidence of the Preservation of Cretaceous Protein. J. Proteome Res..

[20] Abelson P.H. (1955). Paleobiochemistry: Organic constituents of fossils. Carnegie Inst. Wash. Yearb..

[21] Abelson P.H. (1954). Amino acids in fossils. Science.

[22] Tihelka E., Howard R.J., Cai C., Lozano-Fernandez J. (2022). Was There a Cambrian Explosion on Land? The Case of Arthropod Terrestrialization. Biology.

[23] Monson T.A., Brasil M.F., Mahaney M.C., Schmitt C.A., Taylor C.E., Hlusko L.J. (2022). Keeping 21st Century Paleontology Grounded: Quantitative Genetic Analyses and Ancestral State Reconstruction Re-Emphasize the Essentiality of Fossils. Biology.

[24] Torres J.M., Borja C., Gibert L., Ribot F., Olivares E.G. (2022). Twentieth-Century Paleoproteomics: Lessons from Venta Micena Fossils. Biology.

[25] Tahoun M., Engeser M., Namasivayam V., Sander P.M., Müller C.E. (2022). Chemistry and Analysis of Organic Compounds in Dinosaurs. Biology.

[26] López-Antoñanzas R., Mitchell J., Simões T.R., Condamine F.L., Aguilée R., Peláez-Campomanes P., Renaud S., Rolland J., Donoghue P.C.J. (2022). Integrative Phylogenetics: Tools for Palaeontologists to Explore the Tree of Life. Biology.

[27] Tamborini M. (2022). A Plea for a New Synthesis: From Twentieth-Century Paleobiology to Twenty-First-Century Paleontology and Back Again. Biology.

[28] Zhou Z. (2022). The Rising of Paleontology in China: A Century-Long Road. Biology.

[29] Churchill S.E., Keys K., Ross A.H. (2022). Midfacial Morphology and Neandertal–Modern Human Interbreeding. Biology.

[30] Krause J., Briggs A.W., Kircher M., Maricic T., Zwyns N., Derevianko A., Pääbo S. (2010). A Complete mtDNA Genome of an Early Modern Human from Kostenki, Russia. Curr. Biol..

[31] Gokcumen O. (2019). Archaic hominin introgression into modern human genomes. Am. J. Phys. Anthr..

[32] Unterberger S.H., Berger C., Schirmer M., Pallua A.K., Zelger B., Schäfer G., Kremser C., Degenhart G., Spiegl H., Erler S. (2023). Morphological and Tissue Characterization with 3D Reconstruction of a 350-Year-Old Austrian Ardea purpurea Glacier Mummy. Biology.

[33] Tripp M., Wiemann J., Brocks J., Mayer P., Schwark L., Grice K. (2022). Fossil Biomarkers and Biosignatures Preserved in Coprolites Reveal Carnivorous Diets in the Carboniferous Mazon Creek Ecosystem. Biology.

[34] Anné J., Canoville A., Edwards N.P., Schweitzer M.H., Zanno L.E. (2023). Independent Evidence for the Preservation of Endogenous Bone Biochemistry in a Specimen of Tyrannosaurus rex. Biology.

[35] Schweitzer M.H., Zheng W., Zanno L., Werning S., Sugiyama T. (2016). Chemistry supports the identification of gender-specific reproductive tissue in Tyrannosaurus rex. Sci. Rep..

[36] Schweitzer M.H., Wittmeyer J.L., Horner J.R. (2005). Gender-specific reproductive tissue in ratites and Tyrannosaurus rex. Science.

[37] Pessoa-Lima C., Tostes-Figueiredo J., Macedo-Ribeiro N., Hsiou A.S., Muniz F.P., Maulin J.A., Franceschini-Santos V.H., de Sousa F.B., Barbosa F., Line S.R.P. (2022). Structure and Chemical Composition of ca. 10-Million-Year-Old (Late Miocene of Western Amazon) and Present-Day Teeth of Related Species. Biology.

[38] Pakhnevich A., Nikolayev D., Lychagina T. (2022). Crystallographic Texture of the Mineral Matter in the Bivalve Shells of Gryphaea dilatata Sowerby, 1816. Biology.

[39] Heingård M., Sjövall P., Schultz B.P., Sylvestersen R.L., Lindgren J. (2022). Preservation and Taphonomy of Fossil Insects from the Earliest Eocene of Denmark. Biology.

[40] Ullmann P.V., Ash R.D., Scannella J.B. (2022). Taphonomic and Diagenetic Pathways to Protein Preservation, Part II: The Case of Brachylophosaurus canadensis Specimen MOR 2598. Biology.

[41] Ullmann P.V., Macauley K., Ash R.D., Shoup B., Scannella J.B. (2021). Taphonomic and diagenetic pathways to protein preservation, part I: The case of tyrannosaurus rex specimen mor 1125. Biology.

[42] Voegele K.K., Boles Z.M., Ullmann P.V., Schroeter E.R., Zheng W., Lacovara K.J. (2022). Soft Tissue and Biomolecular Preservation in Vertebrate Fossils from Glauconitic, Shallow Marine Sediments of the Hornerstown Formation, Edelman Fossil Park, New Jersey. Biology.

[43] Schroeter E.R., Ullmann P.V., Macauley K., Ash R.D., Zheng W., Schweitzer M.H., Lacovara K.J. (2022). Soft-Tissue, Rare Earth Element, and Molecular Analyses of Dreadnoughtus schrani, an Exceptionally Complete Titanosaur from Argentina. Biology.

[44] Colleary C., O’Reilly S., Dolocan A., Toyoda J.G., Chu R.K., Tfaily M.M., Hochella M.F., Nesbitt S.J. (2022). Using Macro- and Microscale Preservation in Vertebrate Fossils as Predictors for Molecular Preservation in Fluvial Environments. Biology.

[45] Cirilli O., Machado H., Arroyo-cabrales J., Barr C.I., Davis E., Jass C.N., Jukar A.M., Landry Z., Mar A.H., Pandolfi L. (2022). Evolution of the Family Equidae, Subfamily Equinae, in North, Central and South America, Eurasia and Africa during the Plio-Pleistocene. Biology.

[46] Garralda M.D., Weiner S., Arensburg B., Maureille B., Vandermeersch B. (2022). Dental Paleobiology in a Juvenile Neanderthal (Combe-Grenal, Southwestern France). Biology.

[47] Cleland T.P., Schroeter E.R., Colleary C. (2021). Diagenetiforms: A new term to explain protein changes as a result of diagenesis in paleoproteomics. J. Proteom..

[48] Schweitzer M.H., Zheng W., Equall N. (2022). Environmental Factors Affecting Feather Taphonomy. Biology.

